# Potassium iodide, but not potassium iodate, as a potential protective agent against oxidative damage to membrane lipids in porcine thyroid

**DOI:** 10.1186/1756-6614-6-10

**Published:** 2013-08-30

**Authors:** Magdalena Milczarek, Jan Stepniak, Andrzej Lewinski, Malgorzata Karbownik-Lewinska

**Affiliations:** 1 Department of Oncological Endocrinology, Medical University of Łódź, 7/9 Żeligowski Street, Łódź 90-752, Poland; 2 Department of Endocrinology and Metabolic Diseases, Medical University of Łódź, 281/289 Rzgowska Street, Łódź 93-338, Poland; 3 Polish Mother’s Memorial Hospital – Research Institute, 281/289, Rzgowska Street, Łódź 93-338, Poland

**Keywords:** Potassium iodide, Potassium iodate, Thyroid, Lipid peroxidation, Ferrous ion, Hydrogen peroxide

## Abstract

**Background:**

Fenton reaction (Fe^2+^+H_2_O_2_→Fe^3+^+^•^OH+OH^−^) is of special significance in the thyroid gland, as both its substrates, i.e. H_2_O_2_ and Fe^2+^, are required for thyroid hormone synthesis. Also iodine, an essential element supplied by the diet, is indispensable for thyroid hormone synthesis. It is well known that iodine affects red-ox balance. One of the most frequently examined oxidative processes is lipid peroxidation (LPO), which results from oxidative damage to membrane lipids. Fenton reaction is used to experimentally induce lipid peroxidation. The aim of the study was to evaluate effects of iodine, used as potassium iodide (KI) or potassium iodate (KIO_3_), on lipid peroxidation in porcine thyroid homogenates under basal conditions and in the presence of Fenton reaction substrates.

**Methods:**

Porcine thyroid homogenates were incubated in the presence of either KI (0.00005 – 500 mM) or KIO_3_ (0.00005 – 200 mM), without or with addition of FeSO_4_ (30 μM) + H_2_O_2_ (0.5 mM). Concentration of malondialdehyde + 4-hydroxyalkenals (MDA + 4-HDA) was measured spectrophotometrically, as an index of lipid peroxidation.

**Results:**

Potassium iodide, only when used in the highest concentrations (≥50 mM), increased lipid peroxidation in concentration-dependent manner. In the middle range of concentrations (5.0; 10; 25; 50 and 100 mM) KI reduced Fenton reaction-induced lipid peroxidation, with the strongest protective effect observed for the concentration of 25 mM. Potassium iodate increased lipid peroxidation in concentrations ≥2.5 mM. The damaging effect of KIO_3_ increased gradually from the concentration of 2.5 mM to 10 mM. The strongest damaging effect was observed at the KIO_3_ concentration of 10 mM, corresponding to physiological iodine concentration in the thyroid. Potassium iodate in concentrations of 5–200 mM enhanced Fenton reaction-induced lipid peroxidation with the strongest damaging effect found again for the concentration of 10 mM.

**Conclusions:**

Potassium iodide, used in doses generally recommended in iodide prophylaxis, may prevent oxidative damage to membrane lipids in this gland. Toxic effects of iodide overload may result from its prooxidative action. Potassium iodate does not possess any direct beneficial effects on oxidative damage to membrane lipids in the thyroid, which constitutes an additional argument against its utility in iodine prophylaxis.

## Background

Iodine, an essential trace element, is indispensable for thyroid hormone synthesis in humans and animals [1]. The only natural source of iodine is the diet. However, in numerous areas in the world iodine supply from natural sources is inadequate, resulting in iodine deficiency disorders (IDD) [2,3]. Precisely elaborated programs of iodine prophylaxis were introduced in different countries to prevent IDD [4]. These programs are mainly based on salt iodization with the use of either potassium iodide (KI) or potassium iodate (KIO_3_).

These two compounds are characterised by different chemical properties and some differencies in potential toxicity/safety. Iodate is more stable, as iodide is readily oxidized to iodine and lost by evaporation [5]. Whereas reliable methods are validated for quantifying KIO_3_ salt content, further validation is required for countries that use KI in salt iodization programs [6]. All other differences between KIO_3_ and KI suggest the superiority of the latter over the former. First, human iodine bioavailability from KI is higher than from KIO_3_[7,8]. Second, in biofortification of vegetables with iodine, KI was found to be much more effective than KIO_3_[9,10].

In turn, according to some health authorities, the safety of KIO_3_ to humans and animals is not completely documented and in 2002 the French Agency for Food Safety [11] did even question the use of KIO_3_ instead of KI in iodine prophylaxis. Therefore, several experimental studies have been performed to clarify this issue. For example, it has been shown in experimental studies that KIO_3_ does not reveal genotoxic effects [12]. Also comparative studies of the oxidative properties of iodate and other halogenate salts, such as bromate and chlorate, have shown that iodate would be of low, if any, genotoxic potential [13]. It is worth mentioning that both, KI and KIO_3_, reveal similar effectiveness as blockers of radioiodine uptake by the thyroid in rats [14].

Nevertheless, according to similar effectiveness in iodine prophylaxis, both KI and KIO_3_ were initially proved to be used for fortifying salt by the Joint WHO/FAO Expert Committee on Food Additives and Contaminants [15] and they are, along with other iodine salts, still admitted to be added to foods, including food supplements [16].

It is well known that iodine affects red-ox balance [17]. It is especially known for its excellent antioxidative properties in physiological conditions [18,19]. However, prooxidative effects of iodine were also demonstrated in experimental models. For example, in studies in vivo, iodine – given as iodide – expectedly increased MDA level in the rat thyroid and liver [20], or it increased Schiff’s bases in rat lung and liver [21] and – when given as KIO_3_ – in mice liver [22]. The latter change was accompanied by the increased activities of antioxidative enzymes, such as glutathione peroxidase and superoxide dismutase, but only after longer time (3 months) of iodine exposure [22]. Thus, the balance between anti- and prooxidative effects of iodine depends on different factors, such as iodine dose/concentration or the time of action.

However, anti- or prooxidative effects of iodine on the thyroid gland with relation to iodine source, i.e. a chemical compound containing iodine, have never been examined in vitro, thus under conditions reflecting direct effects of these compounds on thyroid follicular cells.

Fenton reaction (Fe^2+^+H_2_O_2_→Fe^3+^+^•^OH+OH^−^), being the basic reaction of oxidative stress, is of special significance in the thyroid gland, as both its substrates, i.e. H_2_O_2_ and Fe^2+^, are required for thyroid hormone synthesis [23]. One of the most frequently examined oxidative processes is lipid peroxidation (LPO), which results from oxidative damage to membrane lipids. Bivalent iron (Fe^2+^) and H_2_O_2_, which initiate Fenton reaction, have been frequently used to experimentally induce lipid peroxidation in different tissues [24-29], the thyroid gland included [30,31]. Also oxidative damage to nuclear and mitochondrial DNA has been induced by Fenton reaction substrates [32].

The aim of the study was to evaluate effects of iodine, used as potassium iodide (KI) or potassium iodate (KIO_3_), on lipid peroxidation in porcine thyroid homogenates under basal conditions and in the presence of Fenton reaction substrates.

Preliminary results of the study were presented (as a poster presentation) during International and European Congress of Endocrinology in 2012 [33].

## Methods

### Ethical approval

The procedures, used in the study, were approved by the Ethics Committee of the Medical University of Lodz, Poland.

### Chemicals

Potassium iodide (KI), potassium iodate (KIO_3_), ferrous sulfate (FeSO_4_) and hydrogen peroxide (H_2_O_2_) were purchased from Sigma (St. Louis, MO). The ALDetect Lipid Peroxidation Assay Kit was obtained from Enzo Life Sciences, Inc. (Zandhoven, Belgium). MilliQ-purified H_2_O was used for preparing all solutions. All the used chemicals were of analytical grade and came from commercial sources.

### Animals

Porcine thyroids were collected from forty five (45) animals at a slaughter-house, frozen on solid CO_2_, and stored at -80°C until assay. Each experiment was repeated three to five times. Therefore, three to five tissue pools were prepared, with nine (9) thyroid glands used for each homogenate pool.

### Assay of lipid peroxidation

Thyroid tissue was homogenized in ice cold 20 mM Tris-HCl buffer (pH = 7.4) (10%, w/v), and then incubated for 30 min at 37°C in the presence of examined substances. Porcine thyroid homogenates were incubated in the presence of either KI (500; 250; 100; 50; 25; 10; 5.0; 2.5; 1.0; 0.5; 0.25; 0.1; 0.05; 0.025; 0.01; 0.005; 0.0025; 0.001; 0.0005; 0.00025; 0.0001; 0.00005 mM) or KIO_3_ (200; 150; 100; 50; 25; 10; 5.0; 2.5; 1.0; 0.5; 0.25; 0.1; 0.05; 0.025; 0.01; 0.005; 0.0025; 0.001; 0.0005; 0.00025; 0.0001; 0.00005 mM) without or with addition of Fenton reaction substrates, i.e. FeSO_4_ (30 μM) + H_2_O_2_ (0.5 mM), or with addition of FeSO_4_ (30 μM) only. According to different solubility of KI and KIO_3_, different highest concentrations of these compounds were used in the study. Eight (8) separate experiments were performed, as it is specified in the Results section. The reactions were stopped by cooling the samples on ice. Each experiment was run in duplicate and repeated three to five times.

### Measurement of lipid peroxidation products

The concentrations of malondialdehyde + 4-hydroxyalkenals (MDA + 4-HDA), as an index of lipid peroxidation, were measured in thyroid homogenates, with the ALDetect Lipid Peroxidation Assay Kit. The homogenates were centrifuged at 3,000 x g for 10 min at 4°C. After obtaining supernatant, each experiment was carried out in duplicate. The supernatant (200 μl) was mixed with 650 μl of a methanol:acetonitrile (1:3, v/v) solution, containing a chromogenic reagent, N-methyl-2-phenylindole, and vortexed. Following the addition of 150 μl of methanesulfonic acid (15.4 M), the incubation was carried out at 45°C for 40 min. The reaction between MDA + 4-HDA and N-methyl-2-phenylindole yields a chromophore, which is spectrophotometrically measured at the absorbance of 586 nm, using a solution of 10 mM 4-hydroxynonenal as the standard. The level of lipid peroxidation is expressed as the amount of MDA + 4-HDA (nmol) per mg protein. Protein was measured, using Bradford’s method [34], with bovine albumin as the standard.

### Statistical analyses

Results are expressed as means ± SE. The data were statistically analyzed, using a one-way analysis of variance (ANOVA), followed by the Student-Neuman-Keuls’ test. The level of p < 0.05 was accepted as statistically significant.

## Results

Two examined substances, i.e. KI and KIO_3_, did not reveal the same effects on oxidative damage to membrane lipids in porcine thyroid homogenates, both under basal conditions and in the presence of Fenton reaction substrates.

**Figure 1:**
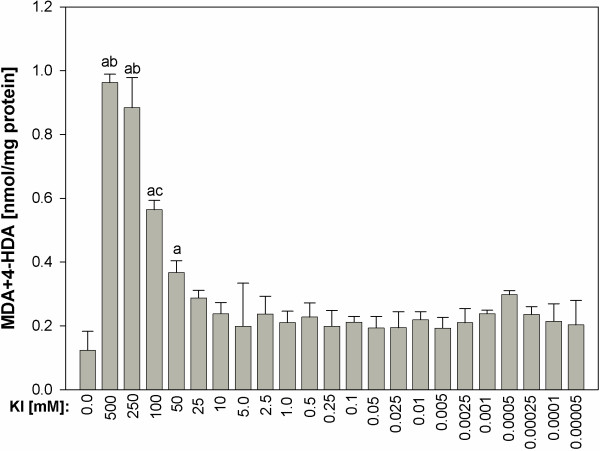
**Lipid peroxidation, measured as MDA + 4-HDA level, in porcine thyroid homogenates.** Homogenates were incubated in the presence of KI alone [500; 250; 100; 50; 25; 10; 5.0; 2.5; 1.0; 0.5; 0.25; 0.1; 0.05; 0.025; 0.01; 0.005; 0.0025; 0.001; 0.0005; 0.00025; 0.0001; 0.00005 mM]. Data are expressed as nmol/mg protein. Data are from four independent experiments. Values are expressed as mean ± SE (error bars). ^a^p < 0.05 *vs*. control (in the absence of KI); ^b^p < 0.05 *vs*. KI concentrations ≤100 mM; ^c^p < 0.05 *vs*. any other KI concentration.

Potassium iodide, when used in the highest concentrations (≥50 mM), did increase lipid peroxidation in concentration-dependent manner. In turn, KI in concentrations ≤25 mM did not affect lipid peroxidation (Figure 1).

**Figure 2:**
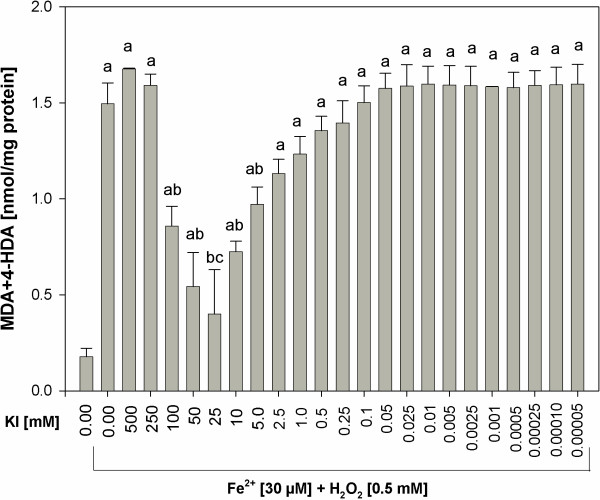
**Lipid peroxidation, measured as MDA + 4-HDA level, in porcine thyroid homogenates.** Homogenates were incubated in the presence of KI [500; 250; 100; 50; 25; 10; 5.0; 2.5; 1.0; 0.5; 0.25; 0.1; 0.05; 0.025; 0.01; 0.005; 0.0025; 0.001; 0.0005; 0.00025; 0.0001; 0.00005 mM] and, additionally, in the presence of both Fenton reaction substrates, namely in the presence of FeSO_4_ [30 μM] plus H_2_O_2_ [0.5 mM]. Data are expressed as nmol/mg protein. Data are from three independent experiments. Values are expressed as mean ± SE (error bars). ^a^p < 0.05 *vs*. control (in the absence of KI or Fe^2+^+H_2_O_2_); ^b^p < 0.05 *vs*. Fe^2+^+H_2_O_2_, *vs*. KI [500 mM] + Fe^2+^+H_2_O_2_, *vs*. KI [250 mM] + Fe^2+^+H_2_O_2_; ^c^p < 0.05 *vs*. KI [100 mM] + Fe^2+^+H_2_O_2_.

When KI was used together with Fe^2+^+H_2_O_2_, the following results were obtained. Potassium iodide used in the middle range of concentrations (5.0; 10; 25; 50 and 100 mM) reduced Fenton reaction-induced lipid peroxidation, with the strongest protective effect observed for the concentration of 25 mM, which completely prevented experimentally-induced lipid peroxidation (Figure 2). Interestingly, KI either in higher (≥250 mM) or lower (≤2.5 mM) concentrations did not affect significantly Fenton reaction-induced lipid peroxidation, which means that the level of lipid peroxidation in the presence of KI (≥250 mM or ≤2.5 mM) plus Fe^2+^+H_2_O_2_ was the same as in the presence of Fe^2+^+H_2_O_2_ only (Figure 2).

**Figure 3:**
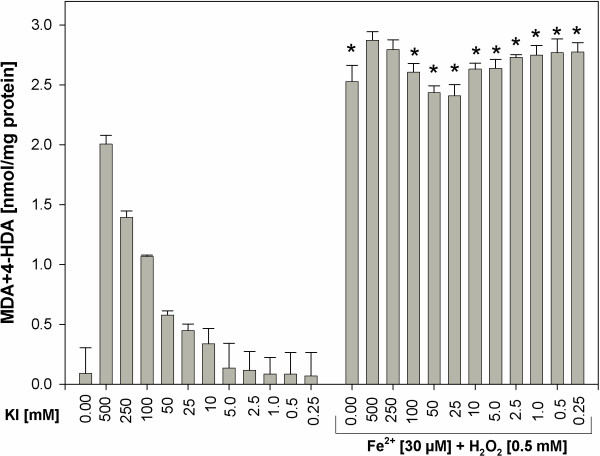
**Lipid peroxidation, measured as MDA + 4-HDA level, in porcine thyroid homogenates.** Homogenates were incubated in the presence of KI alone [500; 250; 100; 50; 25; 10; 5.0; 2.5; 1.0; 0.5; 0.25 mM] or in the presence of KI [500; 250; 100; 50; 25; 10; 5.0; 2.5; 1.0; 0.5; 0.25 mM] together with both substrates of Fenton reaction, namely in the presence of FeSO_4_ [30 μM] plus H_2_O_2_ [0.5 mM]. Data are expressed as nmol/mg protein. Data are from three independent experiments. Values are expressed as mean ± SE (error bars). *p < 0.05 *vs*. respective concentration of KI alone (i.e. in the absence of Fe^2+^+H_2_O_2_).

Subsequently, to compare prooxidative effects of KI alone to prooxidative effects of KI plus Fenton reaction substrates, the separate experiment was performed, in which thyroid homogenates were incubated in the presence of KI (in concentrations of 0.25-500 mM) alone or together with Fe^2+^+H_2_O_2_ (Figure 3). The level of lipid peroxidation was significantly higher when KI (in concentrations ≤100 mM) was used together with Fe^2+^+H_2_O_2_. However, KI did not enhanced Fenton reaction-induced lipid peroxidation (Figure 3), which is in agreement with results shown in Figure 2.

**Figure 4:**
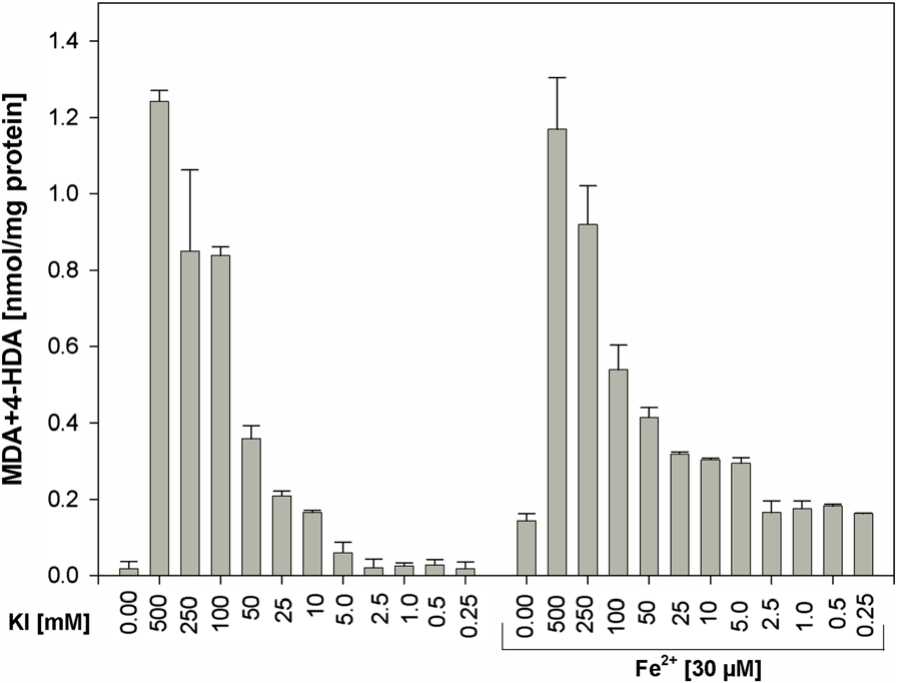
**Lipid peroxidation, measured as MDA + 4-HDA level, in porcine thyroid homogenates.** Homogenates were incubated in the presence of KI alone [500; 250; 100; 50; 25; 10; 5.0; 2.5; 1.0; 0.5; 0.25 mM] or in the presence of KI [500; 250; 100; 50; 25; 10; 5.0; 2.5; 1.0; 0.5; 0.25 mM] together with one substrate of Fenton reaction, namely in the presence of FeSO_4_ [30 μM]. Data are expressed as nmol/mg protein. Data are from three independent experiments. Values are expressed as mean ± SE (error bars). No statistical differences between respective concentrations of KI (i.e. in the presence and in the absence of Fe^2+^) were found.

In our earlier study [31], Fe^2+^ used alone (i.e. as only one of Fenton reaction substrates), in opposite to H_2_O_2_, induced lipid peroxidation in thyroid homogenates. Therefore, in the present study we have performed the additional experiment with use of KI (in concentrations of 0.25-500 mM) alone or together with Fe^2+^ (Figure 4). No significant differences in lipid peroxidation were found for respective concentrations of KI in the presence or in the absence of Fe^2+^ (Figure 4).

**Figure 5:**
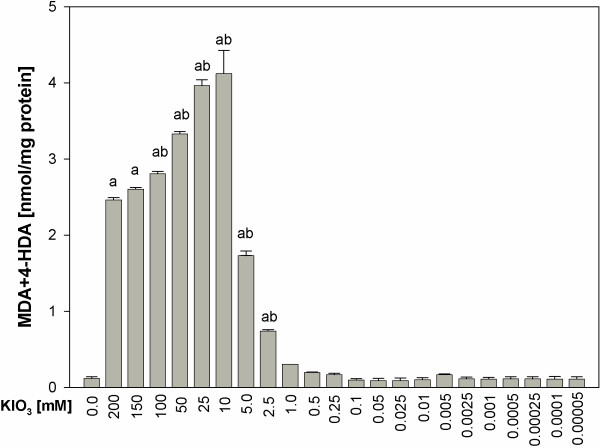
**Lipid peroxidation, measured as MDA + 4-HDA level, in porcine thyroid homogenates.** Homogenates were incubated in the presence of KIO_3_ alone [200; 150; 100; 50; 25; 10; 5.0; 2.5; 1.0; 0.5; 0.25; 0.1; 0.05; 0.025; 0.01; 0.005; 0.0025; 0.001; 0.0005; 0.00025; 0.0001; 0.00005 mM]. Data are expressed as nmol/mg protein. Data are from five independent experiments. Values are expressed as mean ± SE (error bars). ^a^p < 0.05 *vs*. control (in the absence of KIO_3_); ^b^p < 0.05 *vs*. any other KIO_3_ concentration.

**Figure 6:**
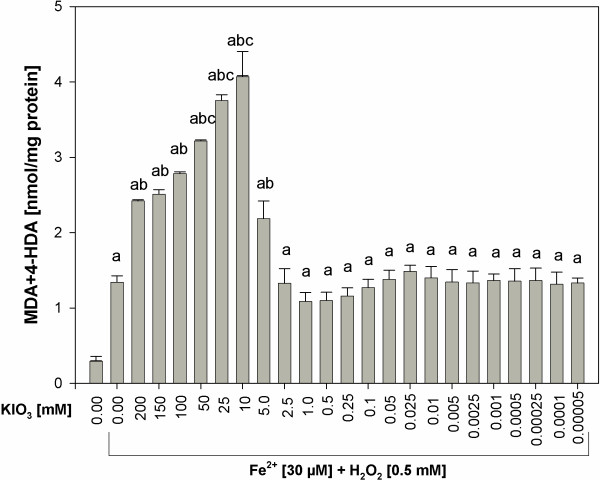
**Lipid peroxidation, measured as MDA + 4-HDA level, in porcine thyroid homogenates.** Homogenates were incubated in the presence of KIO_3_ [200; 150; 100; 50; 25; 10; 5.0; 2.5; 1.0; 0.5; 0.25; 0.1; 0.05; 0.025; 0.01; 0.005; 0.0025; 0.001; 0.0005; 0.00025; 0.0001; 0.00005 mM] and, additionally, in the presence of both Fenton reaction substrates, namely in the presence of FeSO_4_ [30 μM] plus H_2_O_2_ [0.5 mM]. Data are expressed as nmol/mg protein. Data are from five independent experiments. Values are expressed as mean ± SE (error bars). ^a^p < 0.05 *vs*. control (in the absence of KIO_3_ or Fe^2+^+H_2_O_2_); ^b^p < 0.05 *vs*. Fe^2+^+H_2_O_2_ (in the absence of KIO_3_); ^c^p < 0.05 *vs*. any other concentration of KIO_3_.

In the opposite to KI, KIO_3_ revealed, depending on the concentration or the presence/absence of Fe^2+^+H_2_O_2_, either no protective effect at all or even strong prooxidative action (Figures 5 and 6). When KIO_3_ was used alone, it did increase lipid peroxidation in concentrations ≥2.5 mM (Figure 5). The damaging effect of KIO_3_ increased gradually from the concentration of 2.5 mM to 10 mM and, then, it decreased again gradually, however still being significantly stronger at the highest used KIO_3_ concentration (200 mM) than in control (Figure 5). The strongest damaging effect to membrane lipids was observed at the KIO_3_ concentration of 10 mM (Figure 5).

When KIO_3_ was used together with Fe^2+^+H_2_O_2_, the following results were obtained. Potassium iodate enhanced Fenton reaction-induced lipid peroxidation, when it was used in concentrations of 5 mM to 200 mM, with the strongest damaging effect of KIO_3_ found again for the concentration of 10 mM (Figure 6). Concentration-dependent effects of KIO_3_ used together with Fenton reaction substrates were similar to those caused by KIO_3_ alone (compare Figures 5 and 6).

**Figure 7:**
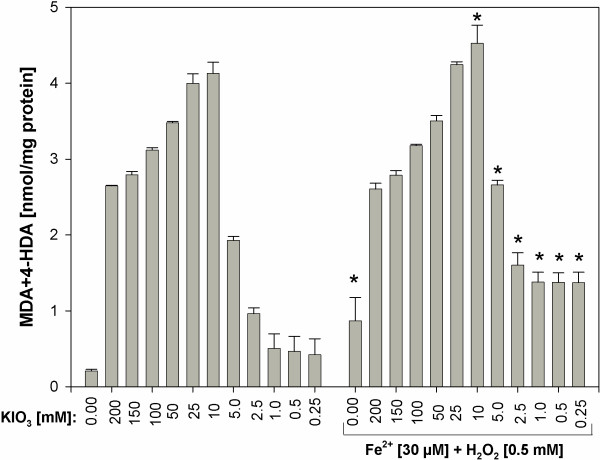
**Lipid peroxidation, measured as MDA + 4-HDA level, in porcine thyroid homogenates.** Homogenates were incubated in the presence of KIO_3_ alone [200; 150; 100; 50; 25; 10; 5.0; 2.5; 1.0; 0.5; 0.25 mM] or in the presence of KIO_3_ [200; 150; 100; 50; 25; 10; 5.0; 2.5; 1.0; 0.5; 0.25 mM] together with both substrates of Fenton reaction, namely in the presence of FeSO_4_ [30 μM] plus H_2_O_2_ [0.5 mM]. Data are expressed as nmol/mg protein. Data are from three independent experiments. Values are expressed as mean ± SE (error bars). *p < 0.05 *vs*. respective concentration of KIO_3_ alone (i.e. in the absence of Fe^2+^+H_2_O_2_).

In comparative experiment (i.e. in the presence and in the absence of Fenton reaction substrates), the addition of Fe^2+^+H_2_O_2_ enhanced KIO_3_-induced lipid peroxidation, but only for KIO_3_ concentration ≤10 mM) (Figure 7). Strong prooxidative effects of KIO_3_ in concentrations ≥25 mM was not enhanced by Fenton reaction substrates (Figure 7).

**Figure 8:**
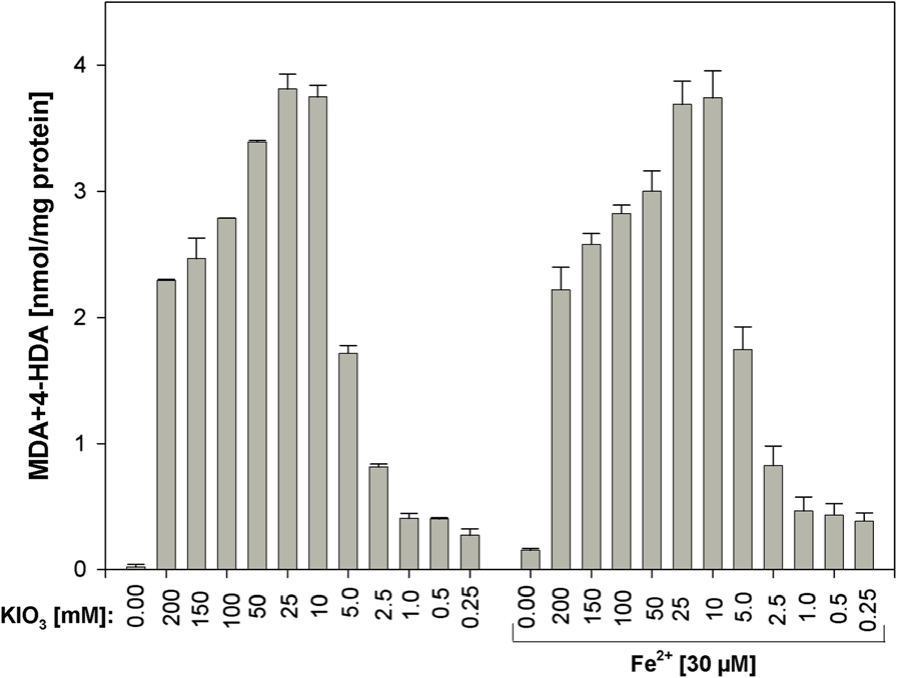
**Lipid peroxidation, measured as MDA + 4-HDA level, in porcine thyroid homogenates.** Homogenates were incubated in the presence of KIO_3_ alone [200; 150; 100; 50; 25; 10; 5.0; 2.5; 1.0; 0.5; 0.25 mM] or in the presence of KIO_3_ [200; 150; 100; 50; 25; 10; 5.0; 2.5; 1.0; 0.5; 0.25 mM] together with one substrate of Fenton reaction, namely in the presence of FeSO_4_ [30 μM]. Data are expressed as nmol/mg protein. Data are from three independent experiments. Values are expressed as mean ± SE (error bars). No statistical differences between respective concentrations of KI (i.e. in the presence and in the absence of Fe^2+^) were found.

In turn, no significant differences were found for respective concentrations of KIO_3_ in the presence or in the absence of Fe^2+^ (Figure 8).

## Discussion

Molar concentrations of KI and KIO_3_ were calculated in the present study with regard to whole compounds. Among chemical elements forming either KI or KIO_3_, iodine is characterized by the highest molecular mass – much higher than potassium (K) and oxygen (O), thus it constitutes the crucial part of both compounds concerning their molecular masses, i.e. iodine constitutes 76.45% of molecular mass of KI, and in case of KIO_3_ it constitutes 59.30% of the molecular mass of this compound. In turn, the molecular mass of KI constitutes 77.57% of that one of KIO_3_, so the molecular masses of these two compounds are of the same order of magnitude. Therefore, concentrations of KI and of KIO_3_, calculated in the present study, may be used in comparative analyses either of effects of iodide ions (I^-^) formed from both KI and KIO_3_, or of effects of the whole compounds, i.e. KI and KIO_3_.

It is worth mentioning that in vitro KI treatment of the thyroid cell line PCCl_3_ resulted in the increased reactive oxygen species production [35]. Similar effect has not been evaluated after KIO_3_ treatment.

Only few studies have been performed till now to compare effects of iodine present in two different sources, namely in KI and KIO_3_. No differences were found between tissue (the thyroid gland, liver, kidney, muscle, abdominal fat tissue and skin) iodine content or blood thyroid hormone concentrations after in vivo treatment with high doses of either KI or KIO_3_[8]. In similar in vivo model the same group of authors evaluated some parameters of oxidative stress [36]. Different compounds of iodine, i.e. KI or KIO_3_, have similar effects on lipid peroxidation level (measured as MDA concentration) in the liver and the muscle, however certain differences were observed concerning mRNA expressions or the activities of antioxidative enzymes in different tissues [36]. However, such comparative studies have not been performed under in vitro conditions. The present study is the first one in which KI was found to be superior to KIO_3_ in the thyroid gland concerning oxidative damage to macromolecules.

More favourable effects of KI comparing to KIO_3_ were observed both, when each of these compounds was used separately or together with Fenton reaction substrates. Concerning the first situation, both KI and KIO_3_ revealed – when used in high concentrations – damaging effects to membrane lipids in the thyroid. However, the difference between these unfavourable actions of KI and KIO_3_ was crucial. Namely, the damaging effect of KI decreased gradually with decreasing concentrations of this compound and these undesired effects were not observed for concentrations below 50 mM, thus concentrations corresponding to inorganic iodine level normally present in the thyroid under physiological conditions. On the basis of experimental findings [37-39] the concentration of inorganic iodine in the human or rat thyroid was calculated by the authors of the present work to be approx. 9 mM. Taking into account close similarity between human and porcine thyroid in terms of thyroid volume and of thyroid hormone synthesis with all elements and all steps of this process [40], it may be estimated that iodine concentration in porcine thyroid (used in the present study) is at similar level. Thus, the concentrations of KI resulting in thyroid level of inorganic iodine close to that observed under physiological conditions do not reveal prooxidative effects, at least in terms of oxidative damage to membrane lipids.

In opposite, when KIO_3_ was used alone, the highest lipid peroxidation was found for its concentration of 10 mM, thus corresponding to the physiological concentration in the thyroid, which was calculated to be 9 mM. It should be recalled that the same concentration of KI did not increase the level of lipid peroxidation (compare Figures 1 and 2).

When KI or KIO_3_ were added to the incubation medium together with Fenton reaction substrates, KI appeared to be also superior to KIO_3_ concerning their influence on lipid peroxidation in the thyroid gland. Whereas KI in concentrations of 5–100 mM (Figure 2), i.e. corresponding to physiological concentrations of inorganic iodine, diminished experimentally-induced lipid peroxidation, KIO_3_ did not reveal any protective action (Figure 5). Additionally, KI in concentration of 25 mM, thus being one order of magnitude higher than physiological iodine concentration, completely prevented Fenton reaction-induced lipid peroxidation. In turn, KIO_3_ enhanced Fenton reaction-induced lipid peroxidation, when it was used in concentrations of 5 mM to 200 mM, with the strongest damaging effect of KIO_3_ found again for the concentration of 10 mM (Figure 6).

Additionally it is clearly visible that prooxidative effects of KI was enhanced by Fenton reaction substrates. At the same time the damaging effects of KIO_3_ were so strong that they were only weakly enhanced by Fenton reaction substrates (compare Figures 1 and 2, Figures 5 and 6, Figures 3 and 7). These comparative analyses reveal additionally the superiority of KI over KIO_3_.

Ferrous ion (Fe^2+^), which was in our earlier study [31] documented to induce lipid peroxidation in the thyroid gland, when it was used as only one of Fenton reaction substrates, did not modify significantly in the present study the effect of either KI or of KIO_3_. This is probably due to the fact that prooxidative effects of Fe^2+^ alone is clearly weaker than those ones caused by both Fenton reaction substrates [31]. Potential mechanisms of differences between KI and KIO_3_ effects on lipid peroxidation in thyroid homogenates, observed in the present study, should be discussed. Thus, the following explanation is proposed.

The reduction of IO_3_^-^, the process occurring when the tissue is exposed to KIO_3_, requires the time and energy and possibly it is associated with unfavorable oxidative reactions and the damaging effects.

In turn, KIO_3_ belongs to halogenate salts, which are known for their potentially toxic effects [5,13,41]. In our earlier studies, one of halogenate salts, namely potassium bromate (KBrO_3_), being classified as carcinogen (group 2B according to IARC 1986 [42]), was shown to exert damaging effect to membrane lipids in porcine thyroid under in vitro (5 mM) and in vivo conditions [43]. Thus, KBrO_3_ and KIO_3_ increased lipid peroxidation in porcine thyroid homogenates when used in the same range of concentrations. At the same time, iodine used as KI (in concentrations ≤25 mM) did not reveal in the present study any toxic effects to membrane lipids and even it prevented experimentally induced lipid peroxidation, when used in the same range of concentrations (5–100 mM).

However, from among three halogenate salts, i.e. iodate, bromate and chlorate, the first one is characterized by the lowest redox potential. Therefore, KIO_3_ seems to be potentially less toxic than bromate and chlorate, at least when oxidative mechanisms are considered.

Our study was performed under in vitro conditions and, therefore, it cannot be extrapolated directly into in vivo conditions, especially that IO_3_^-^ is reduced to I^-^ before approaching the thyroid cell. However, our study is the first one which supports the statement that the use of KI in iodine prophylaxis is more safe than of KIO_3_, in terms of their influence on oxidative damage to macromolecules. Additionally, not only the thyroid gland should be considered in the aspect of prooxidative effects of KIO_3_ but, more importantly, other tissues, in particular digestive system, which is approached by KIO_3_ earlier, i.e. immediately after exposure to this salt.

## Conclusions

Potassium iodide, used in doses generally recommended in iodide prophylaxis, thus resulting in physiological iodine concentration in the thyroid, may prevent oxidative damage to membrane lipids in this gland. Toxic effects of iodide overload may result from its prooxidative action. Potassium iodate does not possess any direct beneficial effects on oxidative damage to membrane lipids in the thyroid, which constitutes an additional argument against its utility in iodine prophylaxis.

## Competing interests

Authors declare that they have no competing interests.

## Authors’ contributions

MM carried out the experiments, performed the statistical evaluation and prepared the draft of the manuscript. JS accompanied the steps of the study related to lipid peroxidation measurement. AL revised the final version of the manuscript. MK-L designed the study, supervised its conducting and prepared the final version of the manuscript. All authors read and approved the final manuscript.
